# Structural insights into the SARS-CoV-2 Omicron RBD-ACE2 interaction

**DOI:** 10.1038/s41422-022-00644-8

**Published:** 2022-04-13

**Authors:** Jun Lan, Xinheng He, Yifei Ren, Ziyi Wang, Huan Zhou, Shilong Fan, Chenyou Zhu, Dongsheng Liu, Bin Shao, Tie-Yan Liu, Qisheng Wang, Linqi Zhang, Jiwan Ge, Tong Wang, Xinquan Wang

**Affiliations:** 1 The Ministry of Education Key Laboratory of Protein Science, Beijing Advanced Innovation Center for Structural Biology, Beijing Frontier Research Center for Biological Structure, Collaborative Innovation Center for Biotherapy, School of Life Sciences, Tsinghua University, Beijing, China; 2 Microsoft Research Asia, Beijing, China; 3 Shanghai Synchrotron Radiation Facility, Shanghai Advanced Research Institute, Chinese Academy of Sciences, Shanghai, China; 4 Department of Chemistry, Tsinghua University, Beijing, China; 5 Center for Global Health and Infectious Diseases, Comprehensive AIDS Research Center, and Beijing Advanced Innovation Center for Structural Biology, School of Medicine, Tsinghua University, Beijing, China

**Keywords:** X-ray crystallography, Molecular biology

Dear Edi﻿tor,

The global fight against the COVID-19 pandemic is still in great uncertainty due to the emergence of SARS-CoV-2 variants, especially the variants of concern (VOCs) with changed pathogenicity, increased transmissibility and the resistance to convalescent/vaccination sera and monoclonal neutralizing antibodies.^1,2^ As a newly reported VOC, the Omicron variant has caused great concern with 32 mutations in the spike glycoprotein including unprecedented 15 mutations in the receptor-binding domain (RBD), which would have significant impact on the viral infectivity and the protection effects of approved vaccines and therapeutic antibodies.^3,4^ A detailed description of the Omicron spike and its interactions with ACE2 receptor and neutralizing antibodies is essential for fully understanding the infection and immune escape of the Omicron variant at the molecular level.

Here we reported the crystal structure of the Omicron RBD-ACE2 complex at 2.6 Å resolution (Supplementary information, Table S1). The overall structure of the Omicron RBD-ACE2 is highly similar to that of the wild-type (WT) RBD-ACE2^5^ (Fig. 1b). There are 15 mutations in the Omicron RBD (G339D, S371L, S373P, S375F, K417N, N440K, G446S, S477N, T478K, E484A, Q493K/R, G496S, Q498R, N501Y, Y505H), with ten in the receptor-binding motif (RBM) for ACE2 binding and five in the core subdomain (Fig. 1a, b). The Omicron RBD in our study contains the Q493K substitution, which was reported at its onset and was recently replaced by Q493R mutation.^6^ Three mutations S371L, S373P and S375F in the core subdomain are clustered at a hairpin loop (residues Y369–C379), resulting in a main-chain conformational change compared to the WT and other mutated RBD structures (Fig. 1c).

**Fig. 1:**
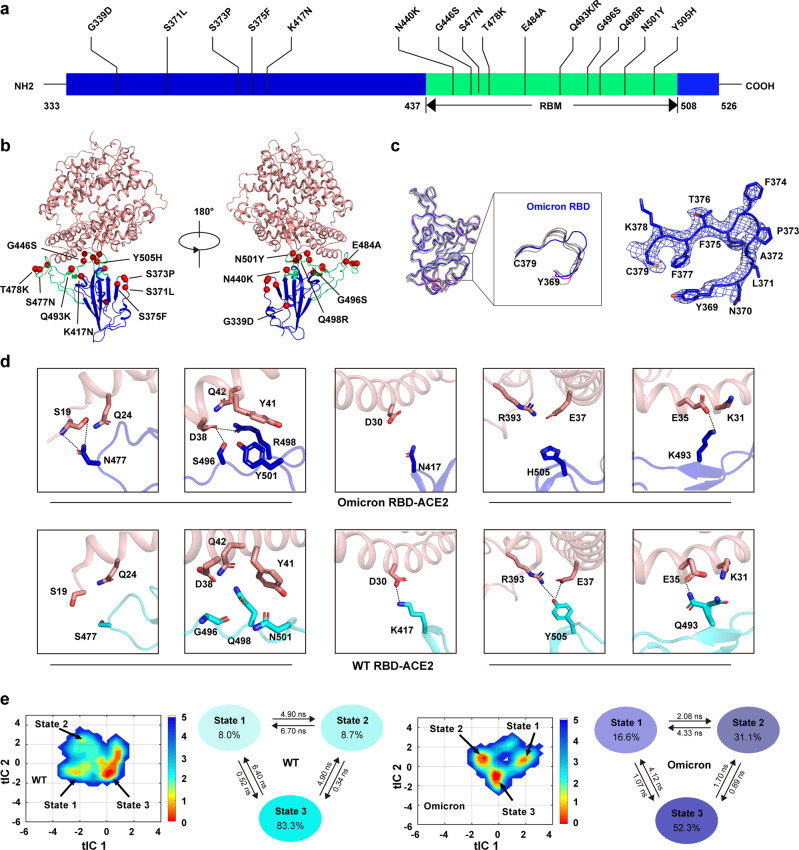
Overall structure of the Omicron RBD bound to ACE2. **a** Substitutions of amino acid residues on the Omicron RBD. **b** Complex structure of the Omicron RBD and ACE2. RBD core subdomain and RBM are shown in blue and green, respectively. ACE2 is shown in salmon. Mutational residues are shown as red sphere. **c** Alignment of the Omicron RBD structure with WT and other mutated RBDs structures previously reported with resolution higher than 3.2 Å (PDB ID 6M0J, 7E7Y, 7NX6, 7NXC and 7R6W). The Omicron RBD is colored in blue. The WT RBD is colored in purple. The other RBDs are shown in gray. 2*F*o-*F*c electron density map of the Omicron RBD hairpin loop (Y369–C379) contoured at 1.0σ is shown. **d** Change of interactions with ACE2 between Omicron RBD (upper panel) and WT RBD (down panel) at the S477N, G496S, Q498R, N501Y, K417N, Y505H and Q493K sites. Omicron RBD, WT RBD and ACE2 are shown in blue, cyan and salmon, respectively. Contacting residues are shown as sticks. Hydrogen bonds or salt bridges are represented by dashed lines. **e** The free energy landscape of WT and Omicron system with the proportion for each state and transition between states. The time-lagged independent component (tIC) 1 and tIC 2 were constructed according to the contacts between residue pairs of RBD and ACE2. The corresponding states are labelled by arrows.

The Omicron and WT RBDs share a highly similar overall binding to the ACE2 receptor. With a distance cut-off of 4 Å, the numbers of contacting residues at the interfaces are similar (Supplementary information, Table S2). However, slight difference in the ACE2-binding affinity was observed between them, and the measured binding affinity between the Omicron RBD and ACE2 is approximately 2.48 ± 1.17 nM, exhibiting a ~2.5-fold increase compared to the WT RBD (*K*_D_ = 6.34 ± 2.19 nM) (Supplementary information, Fig. S1a). The Omicron RBD has a more positive electrostatic potential at the surface for ACE2 binding due to the substitutions to basic residues including N440K, T478K, Q493K and Q498R and the loss of acidic residue E484 (Supplementary information, Fig. S1b).

Detailed structural comparisons revealed that the Omicron RBD obtains newly formed interactions at several sites. One is at the RBD 477 site, in which the WT RBD S477 is not in contact with ACE2, whereas the N477 in the Omicron RBD forms interactions with ACE2 S19 and Q24 (Fig. 1d; Supplementary information, Table S3). A more significant one is around a cluster of RBD 496, 498 and 501 sites. At the WT RBD-ACE2 interface, van del waals interactions are only observed from WT RBD Q498 and N501 to ACE2 Y41 and Q42. Due to the G496S, Q498R and N501Y mutations in the Omicron RBD, newly formed interactions occur between Omicron RBD residues (S496, R498 and Y501) and ACE2 residues (D38, Y41 and Q42). Specifically, S496 forms hydrogen bond with ACE2 D38; R498 forms new hydrogen bond and salt bridge with ACE2 D38; and additional stacking ring interaction is formed between Omicron RBD Y501 and ACE2 Y41 (Fig. 1d; Supplementary information, Table S3). In the meantime, the mutations also result in the loss of interactions between the Omicron RBD and ACE2 in comparison to the WT RBD. No interaction is observed between Omicron RBD N417 and ACE2 D30, whereas salt bridge is formed between WT RBD K417 and ACE2 D30. The Y505H mutation in Omicron RBD disrupts the hydrogen-bonding interactions between WT RBD Y505 and ACE2 E37 and R393 (Fig. 1d; Supplementary information, Table S3). Regarding to the RBD 493 site, the Q493K/R substitution results in newly formed salt bridge with ACE2 E35, while also disrupting previous interactions of Q493 with ACE2 K31 (Fig. 1d; Supplementary information, Table S3). We also utilized Molecular Dynamics (MD) simulation and Molecular Mechanics Generalized Born Surface Area (MM/GBSA) to qualitatively estimate the contributions of each mutation to the binding affinity in Omicron RBD. Four mutations including S477N, G496S, Q498R and N501Y have positive impacts on the binding of the Omicron RBD to ACE2, which is consistent with the above described interaction variations at these four sites (Supplementary information, Table S4).

Furthermore, to explore detailed interaction variations occurred on side chains at the RBD-ACE2 interface between WT and Omicron, we built Markov State Model (MSM), and time-lagged independent component (tIC) analysis showed that WT and Omicron RBD-ACE2 interface contacts have different distributions in the first two components (Fig. 1e). The convergence test on the energy landscape showed that simulation runs have reached convergence and the trajectories have reached equilibrium (Supplementary information, Fig. S2). With a novel lag time chosen algorithm designed by us, three states were identified for WT and Omicron, respectively (Fig. 1e; Supplementary information, Figs. S3, S4) and the differences among these states were found to occur at relative positions of their sidechains, which happened on nanosecond timescale. For both WT and Omicron systems, the similar state 3 is the dominant state and can be quickly reached from the other two states while states 1 and 2 vary from each other (Fig. 1e). As shown in Supplementary information, Figs. S5–S7 and Table S5, detailed structural analysis on MSM states suggested that WT RBD may sample conformations disfavoring ACE2 binding, whereas the mutations including Q493R, G496S, Q498R, N501Y and Y505H in the Omicron RBD have more favorable ACE2 interactions, thereby enhancing its binding to ACE2.

In addition to the impact on ACE2 binding, these mutations in the RBD also enable the Omicron variant to escape from antibody recognition and neutralization. Previous studies showed that most Class I, II and III antibodies lost their neutralizing activities against Omicron variant due to mutations in the epitopes such as K417N and E484A, whereas most Class IV, V and VI antibodies still maintained neutralizing activities.^7^ However, a subset of Class IV antibodies were reported to be sensitive to the G339D, N440K, and G446S mutations in the Omicron RBD.^3^ We paid special attention to the S371L/S373P/S375F mutations within the epitopes of a subset of Class VI antibodies including S2X35, S304, S2A4, H104 and CR3022 (Supplementary information, Fig. S8a–c). Indeed, the significantly reduced neutralization activities have been reported for S2X35 and S304 against the Omicron variant.^8^ Structural studies have revealed hydrogen-bonding interactions occurred between these two antibodies and the hairpin loop (residue Y369–C379) (Supplementary information, Fig. S8d, e). Half of the 16 hydrogen bonds are formed by ACE2 with the RBD residue main-chain atoms and the remaining ones are formed with the side-chain atoms (Supplementary information, Fig. S8d, e). From the perspective of our complex structure, the S371L/S373P/S375F substitutions not only changed the side chains, but also induced a main-chain conformational change, which would disrupt the specific binding of the antibodies to the hairpin loop (residue Y369–C379). Moreover, the binding of S2X35 to the Omicron RBD (*K*_D_ = 2.24 × 10^−8^ M) was reported to decrease by ~100 fold compared to the WT RBD (*K*_D_ = 2.68 × 10^−10^M), which supports the significance of the S371L/S373P/S375F substitutions.^9^ It is expected that antibodies including the hairpin loop in their recognizing epitopes would also be impaired by the Omicron RBD S371L/S373P/S375F substitutions for their binding and neutralization. In consistence with a large number of mutations on the Omicron RBD, the neutralizing ability of serum from convalescent and double-vaccinated individuals was significantly reduced against the Omicron variant.^10^ Fortunately, the third mRNA booster immunization was reported to elicit and maintain highly potent serum neutralizing activity against Omicron.^11^ Further improvement/development of the vaccines and the immunization procedure are still needed for fighting against the Omicron and potential future variants.

When we are submitting and revising this paper, five other reports about the Omicron spike/RBD binding to ACE2 have been formally published.^9,12–15^ To date, four of these structures are available, including two structures solved by cryo-EM (PDB: 7WBL, PDB: 7T9L) and two by crystallography (PDB: 7WBP, PDB: 7NT0). We compared these four structures with ours to uncover that they showed nearly identical binding, with an RMSD of 0.184 Å, 0.897 Å, 0.513 Å and 0.490 Å for 7WBP, 7WBL, 7T9L and 7NT0, respectively (Supplementary information, Fig. S9). Moreover, the MD simulation, binding free energy estimation and MSM in our study further suggested four important mutations (S477N, G496S, Q498R, and N501Y) for the enhanced binding of ACE2 by the Omicron RBD. To the best of our knowledge, this work is the first study that combines structural and computational approaches to provide a dynamic view of detailed side chain perturbations to illustrate the Omicron RBD-ACE2 interactions. In the future study, rigorous and quantitative binding free energy calculations should be performed using more accurate but computational prohibitive approaches such as free energy perturbation.

## Supplementary information

Supplementary Information

## Data Availability

The coordinates and structure factors for the Omicron RBD-ACE2 complex were deposited in Protein Data Bank with accession code 7WHH.
