# Supplementary material for: Structural insights into the SARS-CoV-2 Omicron RBD-ACE2 interaction
Source: Cell Res. 2022 Apr 13;32(6):593–5. doi: 10.1038/s41422-022-00644-8 (PMC9007263; doi:10.1038/s41422-022-00644-8)
Supplement: Supplementary file 1 — Supplementary Information [file 41422_2022_644_MOESM1_ESM.pdf]

Structural insights into the SARS-CoV-2 Omicron RBD-ACE2 interaction

Jun Lan[1,*], Xinheng He[2,*], Yifei Ren[1,*], Ziyi Wang[1,*], Huan Zhou[3], Shilong Fan[1], Chenyou Zhu[4], Dongsheng Liu[4], Bin Shao[2], Tie-Yan Liu[2], Qisheng Wang[3], Linqi Zhang[5,#], Jiwan Ge[1,#], Tong Wang[2,#], Xinquan Wang[1,#]

[1]The Ministry of Education Key Laboratory of Protein Science, Beijing Advanced Innovation Center for Structural Biology, Beijing Frontier Research Center for Biological Structure, Collaborative Innovation Center for Biotherapy, School of Life Sciences, Tsinghua University, Beijing, China

[2]Microsoft Research Asia, Beijing, China.

[3]Shanghai Synchrotron Radiation Facility, Shanghai Advanced Research Institute, Chinese Academy of Sciences, Shanghai, China

[4]Department of Chemistry, Tsinghua University, Beijing, China

[5]Center for Global Health and Infectious Diseases, Comprehensive AIDS Research Center, and Beijing Advanced Innovation Center for Structural Biology, School of Medicine, Tsinghua University, Beijing, China

*These authors contributed equally to this work.

[#]Correspondence: zhanglinqi@mail.tsinghua.edu.cn, (L.Z.), gejw@mail.tsinghua.edu.cn (J. G.), watong@microsoft.com (T.W.), xinquanwang@mail.tsinghua.edu.cn (X.W.)

Supplementary Table 1 | Data collection and refinement statistics

	Omicron RBD - ACE2
**Data collection**	
Space group	$P4_12_12$
Cell dimensions	
a, b, c (Å)	104.71, 104.71, 227.10
α, β, γ (°)	90, 90, 90
Resolution (Å)	50-2.60 (2.66-2.60)
R_{merge}	0.26 (2.45)
I/sI	11.3 (1.3)
Completeness (%)	99.77 (98.58)
Redundancy	14.1 (11.2)
**Refinement**	
Resolution (Å)	36.54-2.60
No. reflections	39525
R_{work}/R_{free}	19.2/23.1
No. atoms	
Protein	6440
Ligand/ion	96
Water	131
B-factors	
Protein	50.47
Ligand/ion	90.06
Water	45.36
R.m.s. deviations	
Bond lengths (Å)	0.008
Bond angles (°)	0.93
Ramachandran	
Favored (%)	96.95
Allowed (%)	3.05
Outliers (%)	0.00

Supplementary Table 2 | Contact residues at the WT and Omicron RBD-ACE2 interfaces

ACE2	WT RBD	Omicron RBD
S19		A475, N477
Q24	A475, N487	A475, N477, N487
T27	F456, A475, Y489	F456, Y489
F28	Y489	Y489
D30	K417, F456	
K31	Y489, Q493	F456, Y489
H34	Y453, L455, Q493	Y453, K493, S494
E35	Q493	K493
E37	Y505	
D38	Y449	Y449, S496, R498
Y41	Q498, T500, N501	R498, T500, Y501
Q42	G446, Y449, Q498	Y449, R498
L79	F486	F486
M82	F486	F486
Y83	F486, N487, Y489	F486, N487, Y489
N330	T500	T500
K353	G496, N501, G502, Y505	Y501, G502, H505
G354	G502	G502, H505
D355	T500	T500
R357	T500	T500
R393	Y505	

A distance cut-off of 4 Å was used.

Supplementary Table 3 | The hydrogen bonds and salt bridges at the WT and Omicron RBD-ACE2 interfaces

	WT RBD	Length(Å)	ACE2	Length(Å)	Omicron RBD
**Hydrogen bonds**			S19(O)	3.2	N477(ND2)
			S19(OG)	3.1	A475(O)
			S19(N)	3.4	N477(OD1)
	N487(ND2)	2.6	Q24(OE1)	2.8	N487(ND2)
	K417(NZ)	3.0	D30(OD2)		
			H34(ND1)	2.9	Y453(OH)
	Q493(NE2)	2.8	E35(OE2)	3.1	K493(NZ)
	Y505(OH)	3.2	E37(OE2)		
			D38(OD1)	2.9	R498(NH1)
			D38(OD1)	2.8	S496(OG)
	Y449(OH)	2.7	D38(OD2)	2.5	Y449(OH)
	T500(OG1)	2.6	Y41(OH)	2.6	T500(OG1)
	N501(N)	3.7	Y41(OH)		
	G446(O)	3.3	Q42(NE2)		
	Y449(OH)	3.0	Q42(NE2)	3.4	Y449(OH)
	Y489(OH)	3.5	Y83(OH)	3.5	Y489(OH)
	N487(OD1)	2.7	Y83(OH)	2.4	N487(OD1)
	G502(N)	2.8	K353(O)	2.7	G502(N)
	Y505(OH)	3.7	R393(NH2)		
**Salt bridges**	K417(NZ)	3.9	D30(OD1)		
	K417(NZ)	3.0	D30(OD2)		
			E35(OE2)	3.1	K493(NZ)
			D38(OD1)	2.9	R498(NH1)
			D38(OD1)	3.7	R498(NH2)

Supplementary Table 4 | The energy decomposition of key interface residues for Omicron and WT RBD in the balanced trajectory

System	Key residues	ΔG (kcal/mol)
WT	S477	-0.56 ± 0.94
WT	Q493	-3.69 ± 2.56
WT	G496	-1.17 ± 1.43
WT	Q498	-2.05 ± 2.61
WT	N501	-1.33 ± 1.07
WT	Y505	-4.10 ± 0.99
WT	Total value	-26.66 ± 9.78
Omicron	N477	-1.33 ± 1.33
Omicron	R493	-2.79 ± 1.94
Omicron	S496	-1.53 ± 1.18
Omicron	R498	-4.91 ± 1.54
Omicron	Y501	-7.17 ± 0.72
Omicron	H505	-3.36 ± 1.85
Omicron	Total value	-42.19 ± 6.61

Supplementary Table 5 | The energy decomposition of key interface residues for Omicron and WT RBD in macrostates

System	Key residues	ΔG (kcal/mol)
WT (state 1)	Y449	-1.85 ± 0.79
WT (state 1)	F486	-3.82 ± 1.32
WT (state 1)	Q493	-2.76 ± 2.22
WT (state 1)	G496	-0.95 ± 1.18
WT (state 1)	Q498	-0.22 ± 0.65
WT (state 1)	N501	-1.14 ± 0.79
WT (state 1)	Y505	-3.64 ± 0.75
WT (state 1)	Total value	-26.25 ± 9.76
WT (state 2)	Y449	-0.27 ± 0.45
WT (state 2)	F486	-1.90 ± 1.67
WT (state 2)	Q493	-2.06 ± 2.59
WT (state 2)	G496	-0.01 ± 0.11
WT (state 2)	Q498	-0.29 ± 0.53
WT (state 2)	N501	-0.75 ± 0.77
WT (state 2)	Y505	-1.98 ± 1.83
WT (state 2)	Total value	-3.88 ± 19.64
WT (state 3)	Y449	-1.80 ± 1.06
WT (state 3)	F486	-3.52 ± 0.83
WT (state 3)	Q493	-4.00 ± 2.20
WT (state 3)	G496	-2.05 ± 1.11
WT (state 3)	Q498	-5.43 ± 2.35
WT (state 3)	N501	-2.61 ± 1.28
WT (state 3)	Y505	-4.39 ± 0.94
WT (state 3)	Total value	-36.20 ± 8.64
Omicron (state 1)	Y449	-1.09 ± 0.68
Omicron (state 1)	F486	-3.03 ± 1.13
Omicron (state 1)	R493	-3.95 ± 1.80
Omicron (state 1)	S496	-1.68 ± 0.87
Omicron (state 1)	R498	-3.71 ± 2.11
Omicron (state 1)	Y501	-7.22 ± 0.94

Omicron (state 1)	H505	-3.71 ± 2.12
Omicron (state 1)	Total value	-40.84 ± 6.93
Omicron (state 2)	Y449	-0.75 ± 0.66
Omicron (state 2)	F486	-3.02 ± 0.76
Omicron (state 2)	R493	-4.13 ± 3.81
Omicron (state 2)	S496	-1.01 ± 1.08
Omicron (state 2)	R498	-2.42 ± 3.08
Omicron (state 2)	Y501	-7.08 ± 1.05
Omicron (state 2)	H505	-4.49 ± 2.34
Omicron (state 2)	Total value	-35.54 ± 8.11
Omicron (state 3)	Y449	-0.93 ± 0.79
Omicron (state 3)	F486	-3.27 ± 0.89
Omicron (state 3)	R493	-3.37 ± 2.01
Omicron (state 3)	S496	-1.21 ± 0.90
Omicron (state 3)	R498	-3.04 ± 3.04
Omicron (state 3)	Y501	-6.88 ± 1.02
Omicron (state 3)	H505	-3.25 ± 1.64
Omicron (state 3)	Total value	-36.41± 10.26

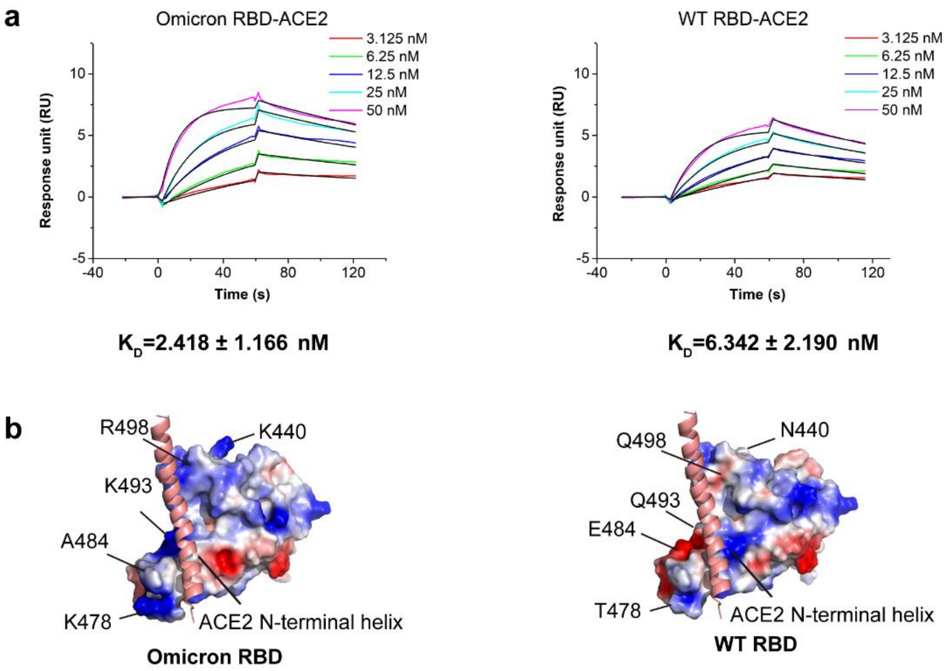

Supplementary Fig. 1 Comparison of the binding to ACE2 between Omicron and WT RBDs. (**a**) SPR curves of ACE2 with Omicron RBD (left) and WT RBD (right). Experimental curves are shown as colored lines and the fitting curves are colored in black. (**b**) Electrostatic potential maps of the Omicron RBD (left) and WT RBD (right). The N-terminal helix is shown in a salmon ribbon. The positions of mutation residues are indicated by a black line.

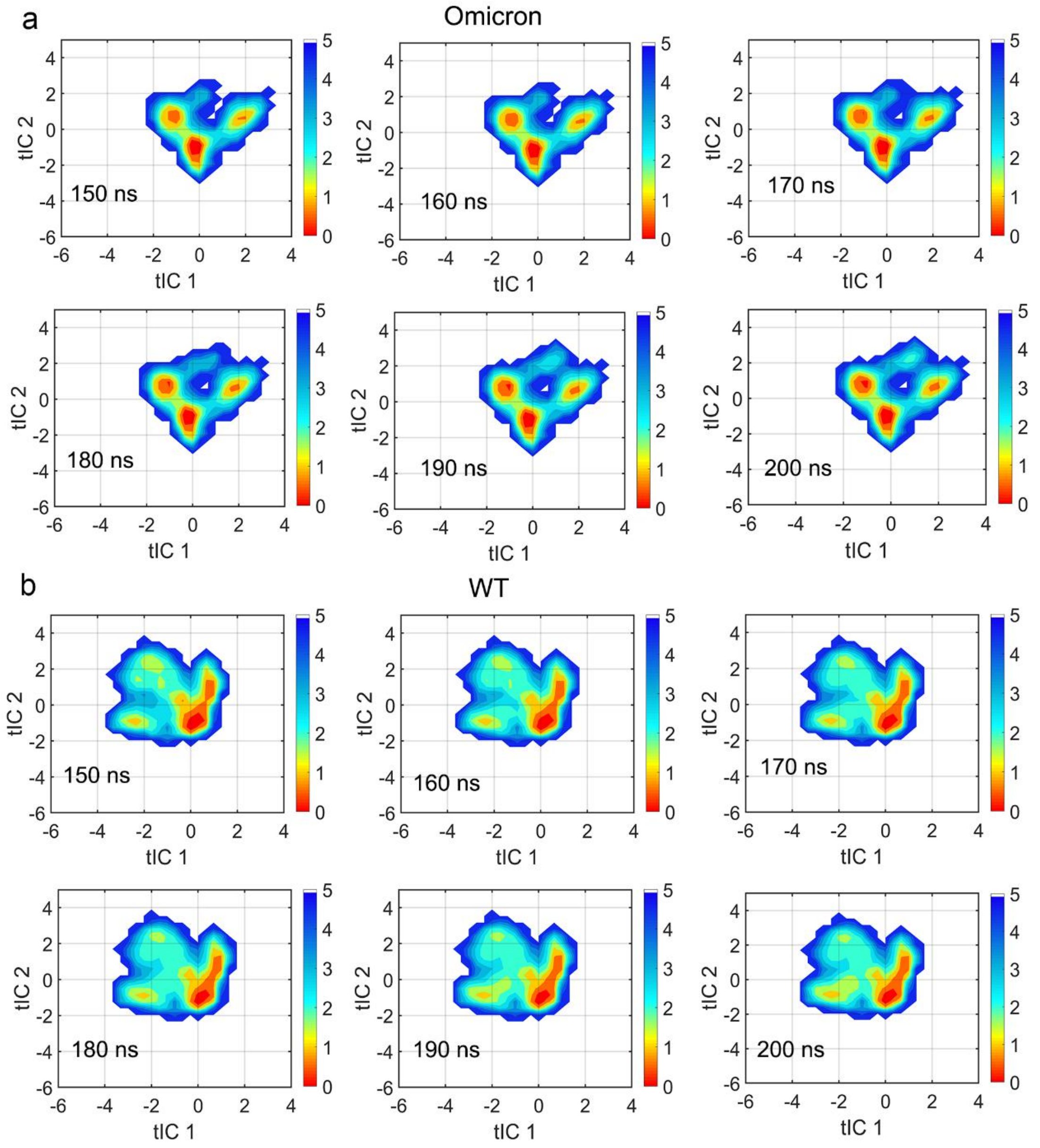

Supplementary Fig. 2 Convergence test on the free energy landscapes for different trajectory length (150, 160, 170, 180, 190, and 200 ns) of Omicron (a) and WT (b). With the same tIC parameters, all subfigures show a highly similar appearance and conformational distribution, confirming that longer simulation time will not sample more conformations on the landscape. Thus, the current trajectory timescale is enough to explore the conformational space of the RBD-ACE2 interface.

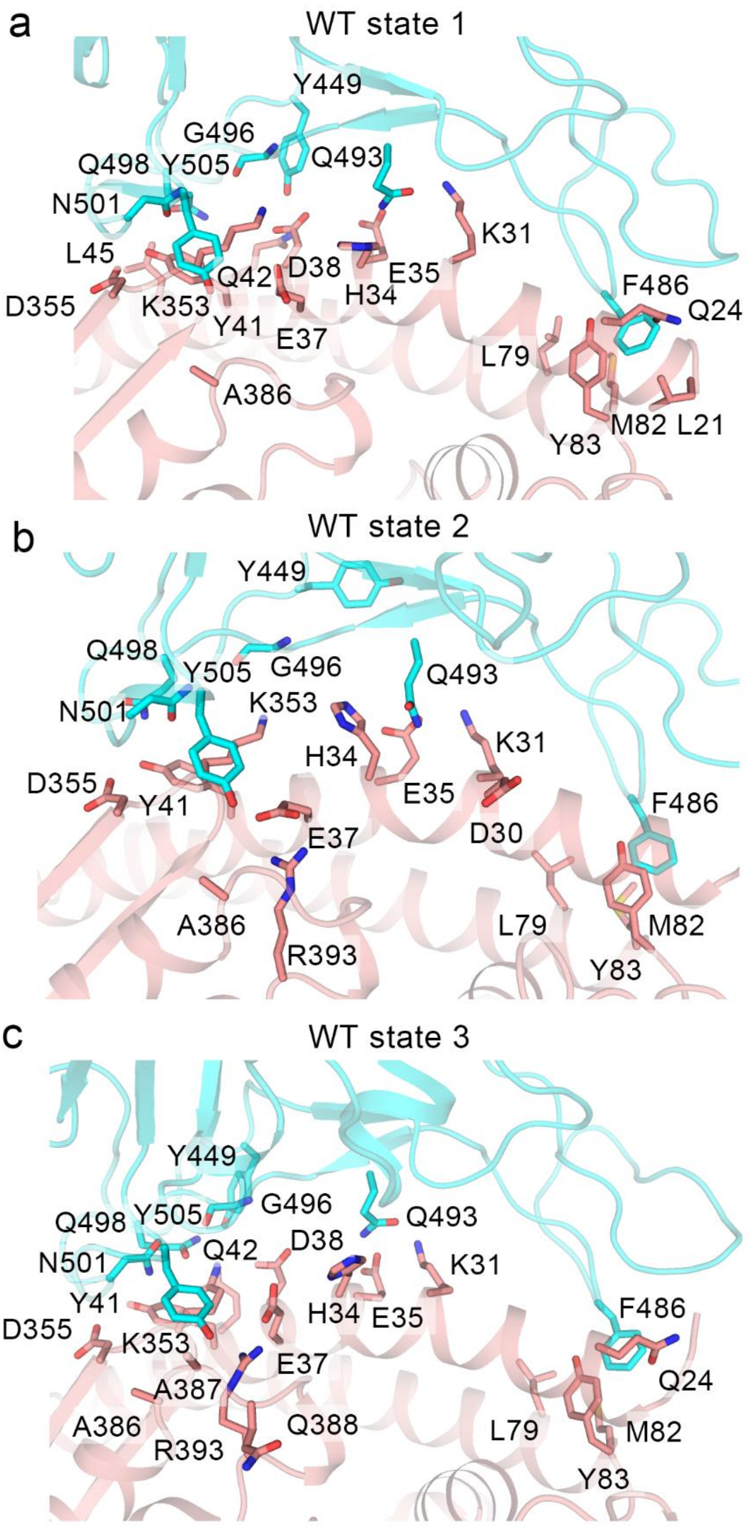

Supplementary Fig. 3 The interactions between ACE2 and RBD in WT state 1 (a), WT state 2 (b) and WT state 3 (c). The residues in ACE2 within 5 Å to Y449, F486, Q493, G496, Q498, N501, and Y505 are shown in sticks. The RBD of WT and ACE2 are shown in cyan and salmon, respectively.

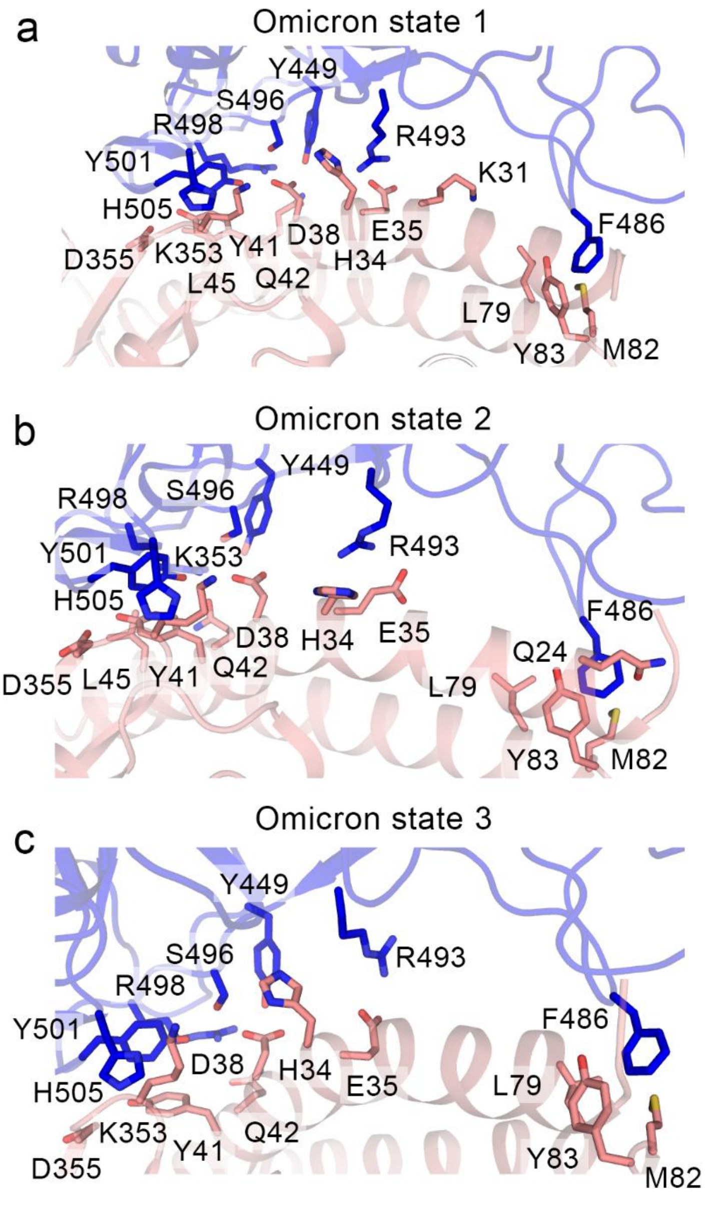

Supplementary Fig. 4 The interactions between ACE2 and RBD in Omicron state 1 (a), Omicron state 2 (b) and Omicron state 3 (c). The residues in ACE2 within 5 Å to Y449, F486, R493, S496, R498, Y501, and H505 are shown in sticks. The RBD of Omicron and ACE2 are shown in blue and salmon, respectively.

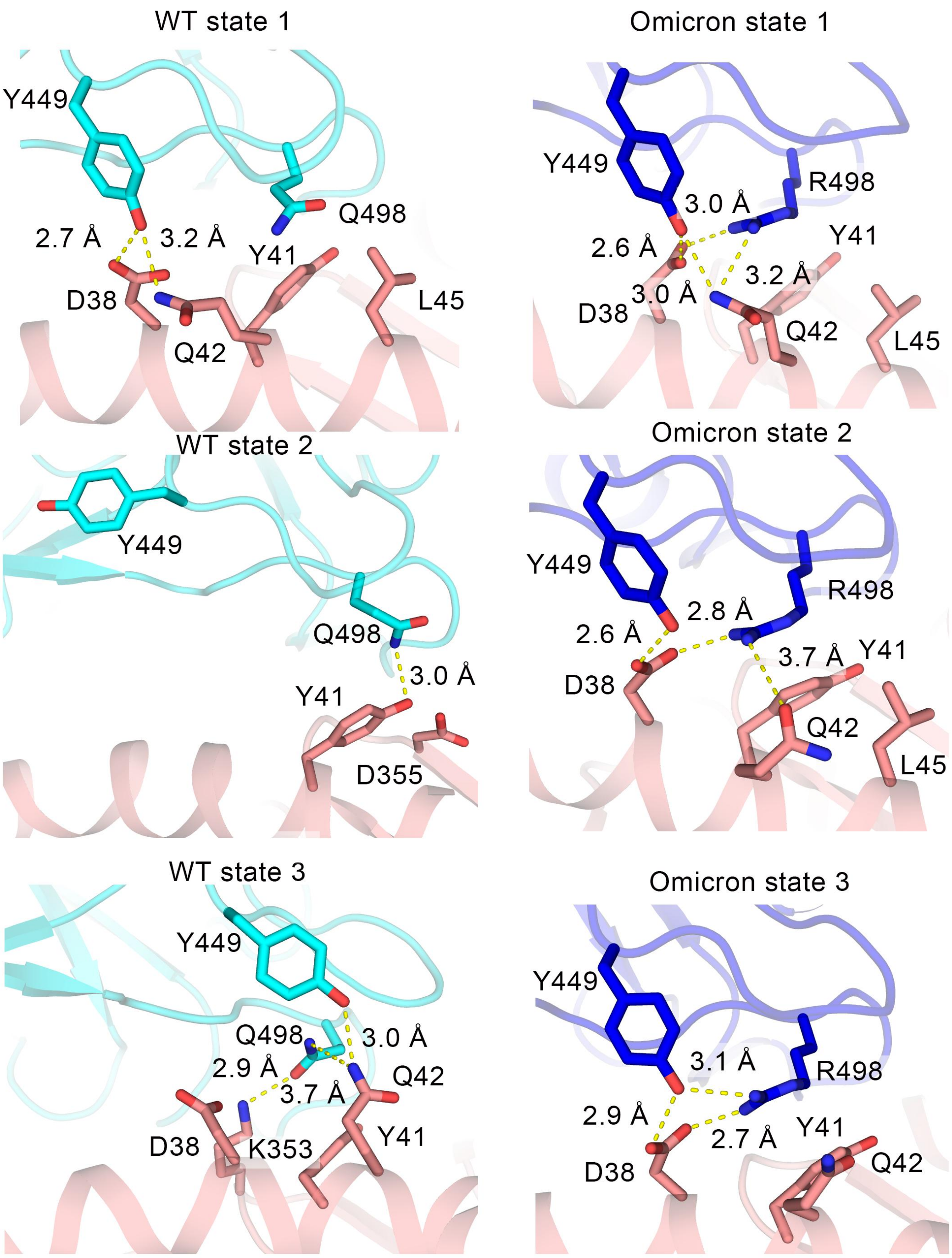

Supplementary Fig. 5 The interactions between Y449 and Q/R498 of RBD with ACE2 in WT and Omicron systems. The residues in ACE2 within 5 Å to Y449 and Q/R498 are shown in sticks while interactions are shown in yellow dashed lines. The RBD of WT, the RBD of Omicron and ACE2 are shown in cyan, blue and salmon, respectively. In WT system, Y449 shows a unique horizontal pose in state 2 which is unseen in other states. The loss of interactions with D38 and Q42 may lead to the decrease of Y449 contribution in WT state 2. In the Omicron system, however, the hydrogen bond network among D38, Q42 Y449 and R498 reduces the possibility of

the horizontal conformation of Y449 and also promotes the interactions between R498 and ACE2. In WT state 3, Q498 interacts with Q42, which similarly promotes its binding affinity (ΔG = -5.43 ± 2.35 kcal/mol).

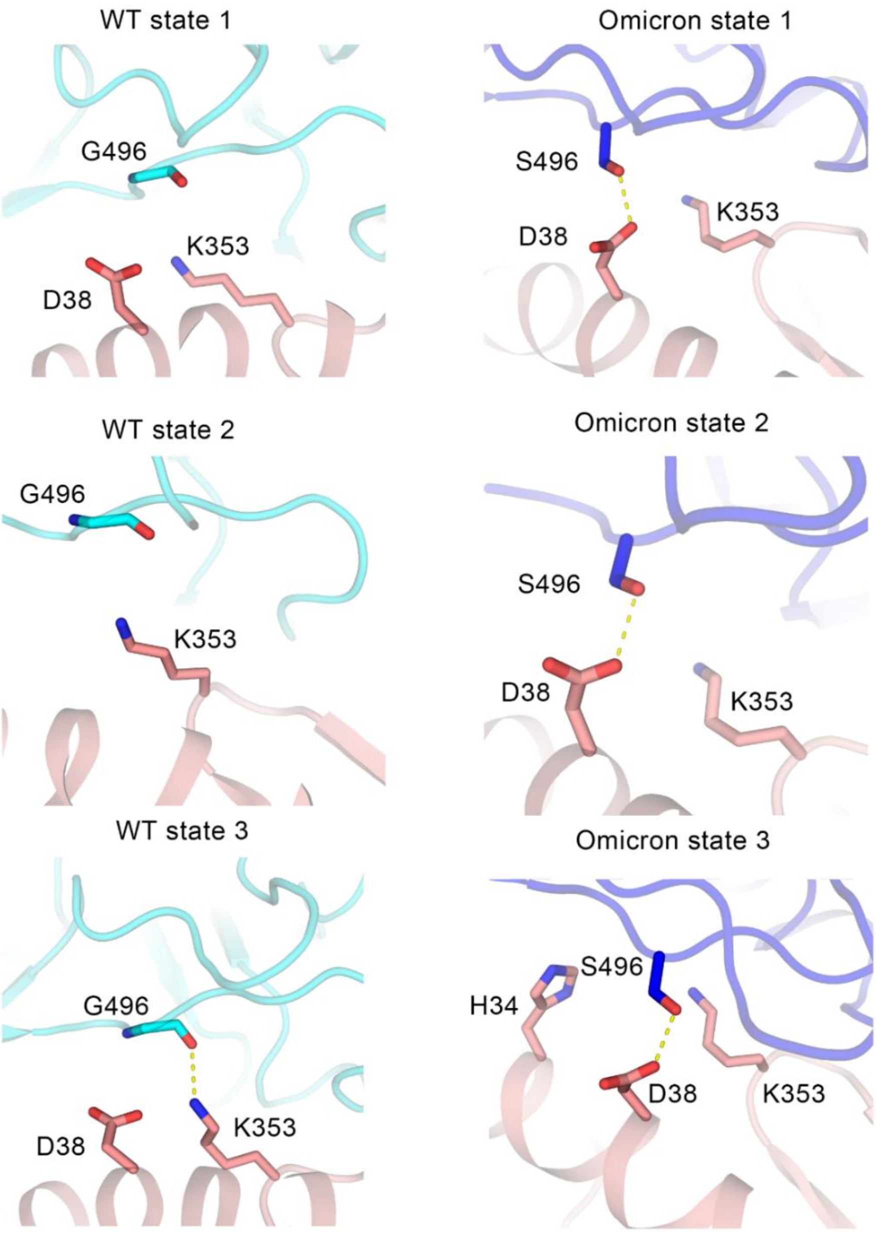

Supplementary Fig. 6 The interactions between G/S496 of RBD with ACE2 in WT and Omicron systems. The residues in ACE2 within 5 Å to G/S496 are shown in sticks while interactions are shown in yellow dashed lines. The RBD of WT, the RBD of Omicron and ACE2 are shown in cyan, blue and salmon, respectively. In WT system, G496 in state 1 and state 2 show no interaction with ACE2, leading to weak contribution to the binding affinity (-0.95 ± 1.18 and -0.01 ± 0.11 kcal/mol, respectively). As for state 3, G496 interacts with K353 in its main chain, which leads

to an increase of the binding ability (-2.05 ± 1.11 kcal/mol). As a contrast, in Omicron system, S496 interacts with D38 in all three macrostates and behaves a lower binding free energy.

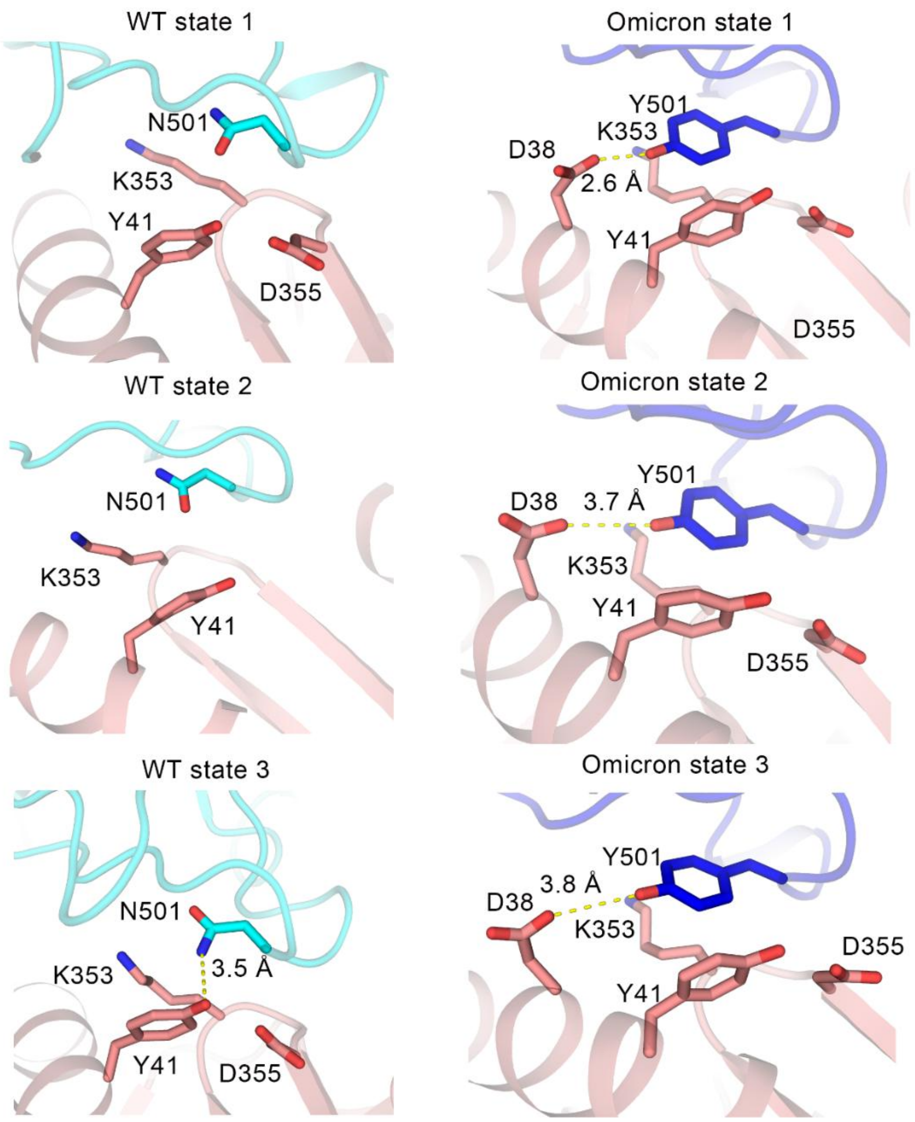

Supplementary Fig. 7 The interactions between N/Y501 of RBD with ACE2 in WT and Omicron systems. The residues in ACE2 within 5 Å to N/Y501 are shown in sticks while interactions are shown in yellow dashed lines. The RBD of WT, the RBD of Omicron and ACE2 are shown in cyan, blue and salmon, respectively. In WT system, N501 also has no interaction with ACE2 in states 1 and 2 and shows weak energy contribution (-1.14 ± 0.79 and -0.74 ± 0.77 kcal/mol, respectively). Even in WT state 3, the energy does not decrease too much with the hydrogen bond to Y41 (-2.61 ± 1.28 kcal/mol). This phenomenon may be caused by the hydrophilic sidechain of N501 embedded in a hydrophobic environment made by the sidechains

of K353 and Y51. As N501Y changes to a residue with a longer sidechain and a hydrophobic phenyl ring in Omicron system, the interaction with D38 is stable in each macrostate and the ring of tyrosine is suitable in the hydrophobic environment.

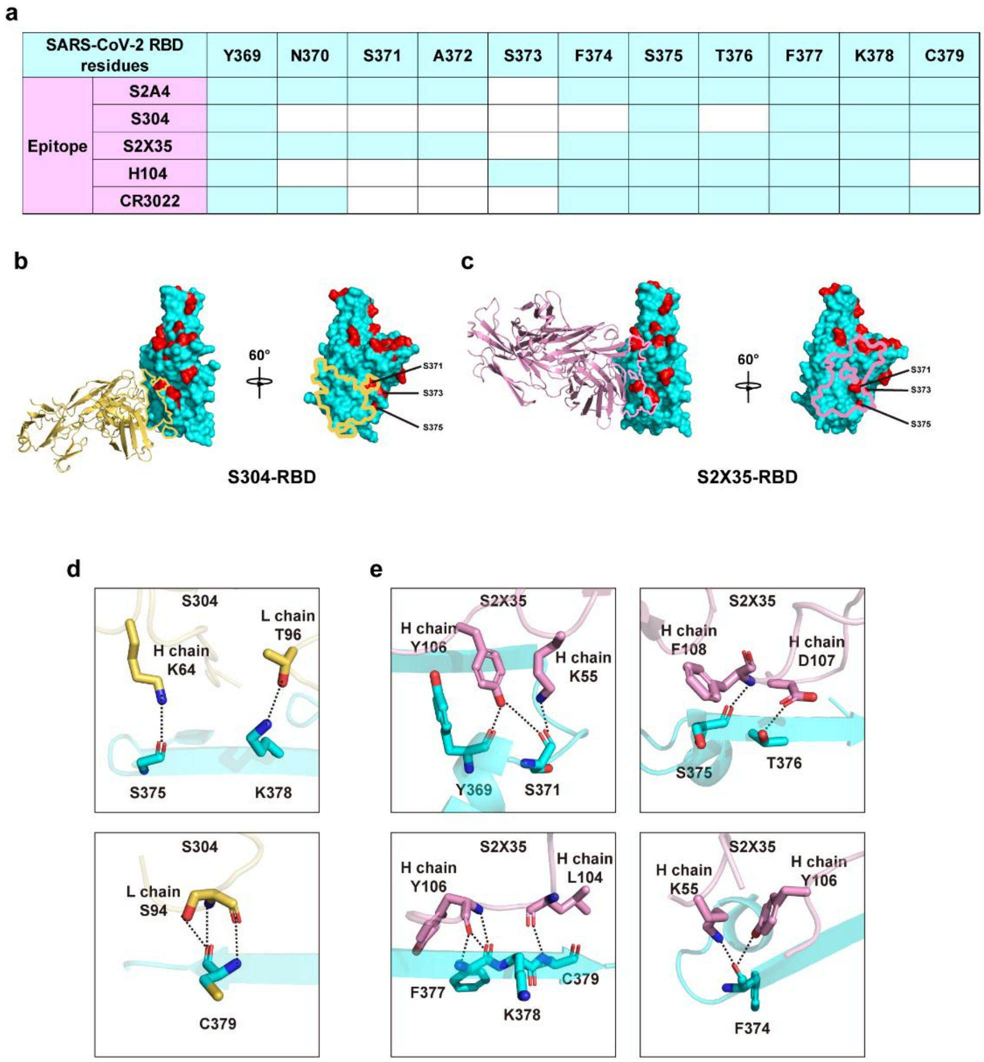

Supplementary Fig. 8 Structural basis of S371L/S373P/S375F mutations for escaping the neutralization of Class VI antibodies. (**a**) The binding residues of Class VI antibodies (S2A4, S304, S2X35, H104 and CR3022) at the hairpin loop. The residues recognized by antibodies is labeled with cyan box. (**b, c**) The binding mode and epitopes of representative Class VI antibodies on RBD; S304 Fab-RBD complex (**b**) and S2X35 Fab-RBD complex (**c**) are shown, with RBD shown as cyan surface. S304 Fab and S2X35 Fab is shown as cartoon with yellow and pink, respectively. The epitope of S304 and S2X35 on RBD is shown with yellow and pink lines, respectively. The mutations of Omicron variant are shown with red surface. (**d, e**) Interactions between the S304 (**d**) or S2X35 (**e**) and SARS-CoV-2 RBD. The hydrogen bonds are displayed with black dashed lines.

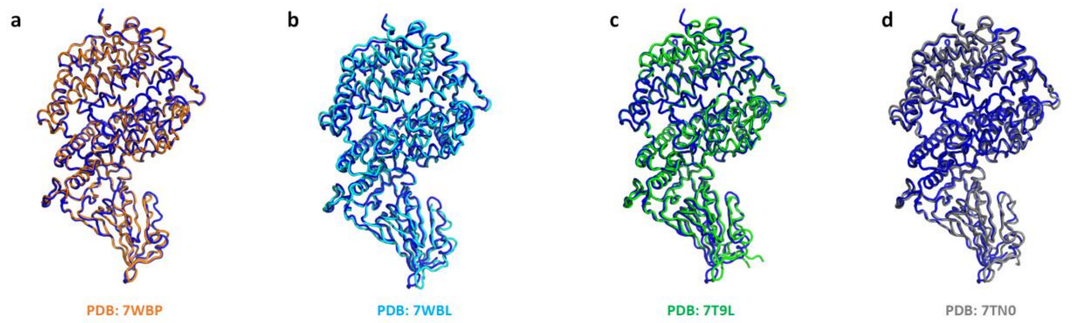

Supplementary Fig. 9 Overall structure alignment of Omicron RBD/ACE2. (a-d) Other published Omicron RBD/ACE2 structures (PDB ID 7WBP, 7WBL, 7T9L and 7TN0) is aligned to ours respectively. Our structure is colored in blue and coordinate of 7WBP, 7WBL, 7T9L and 7TN0 is colored in orange, cyan, green and gray, respectively.

Methods

Protein expression and purification

The SARS-CoV-2 Omicron RBD and the N-terminal peptidase domain of ACE2 were expressed using the Bac-to-Bac baculovirus system (Invitrogen) as previously stated. Briefly, The SARS-CoV-2 Omicron RBD (residues Thr333–Gly526) with an N-terminal gp67 signal peptide for secretion and a C-terminal 6×His tag for purification was expressed using Hi5 cells and purified by Ni-NTA resin and gel filtration chromatography (GE Healthcare) in HBS buffer (10 mM HEPES, pH 7.2, 150 mM NaCl).

The N-terminal peptidase domain of ACE2 (residues Ser19–Asp615) was expressed and purified by essentially the same protocol as used for the SARS-CoV-2 Omicron RBD. To obtain the SARS-CoV-2 Omicron RBD and human ACE2 complex, ACE2 was incubated with the SARS-CoV-2 Omicron RBD for 1 h on ice in HBS buffer, and the mixture was then subjected to gel filtration chromatography. Fractions containing the complex were pooled and concentrated to 13 mg ml^{-1}.

Crystallization and data collection

Crystals were successfully grown at room temperature in sitting drops, over wells containing 0.2 M L-Proline, 0.1 M HEPES pH 7.5, 10% w/v Polyethylene glycol 3,350. Crystals were collected, soaked briefly in 0.2 M L-Proline, 0.1 M HEPES pH 7.5, 10% w/v Polyethylene glycol 3,350 and 20% glycerol, and were subsequently flash-frozen in liquid nitrogen. Diffraction data were collected at 100 K and at a wavelength of 1.07180 Å at the BL02U1 beam line of the Shanghai Synchrotron Research Facility. Diffraction data were processed using the HKL3000 software[1] and the data-processing statistics are listed in Extended Data Table 1.

Structure determination and refinement

The structure was determined using the molecular replacement method with PHASER in the CCP4 suite[2]. The search models used included the ACE2 extracellular domain and SARS-CoV-2 RBD (PDB: 6M0J). Subsequent model building and refinement were performed using COOT[3] and PHENIX[4], respectively. Final Ramachandran statistics: 96.95% favored, 3.05% allowed and 0.00% outliers for the final structure. The structure refinement statistics are listed in Extended Data Table 1. All structure figures were generated with PyMol[5].

Surface plasmon resonance

Binding kinetics of ACE2 and SARS-CoV-2 RBDs were determined by surface plasmon resonance using a Biacore S200 (GE Healthcare). All experiments were performed in a running

buffer composed of 10 mM HEPES, pH 7.2, 150 mM NaCl, and 0.005% Tween-20 (v/v). ACE2 was immobilized on a CM5 sensor chip (GE Healthcare) to a level of ~500 response units. A 2-fold dilution series ranging from 50 to 3.125 nM of the SARS-CoV-2 WT RBD and omicron RBD were injected at a flow rate of 30 μl/min (association 60s, dissociation 180s), and the immobilized ACE2 was regenerated using 5mM NaOH for 10s. The resulting data were fit to a 1:1 binding model using Biacore Evaluation Software (GE Healthcare).

MD simulations

The Omicron RBD-ACE2 structure and WT RBD-ACE2 complex (PDB ID: 6M0J) were used to build the MD simulation systems. Besides, K493 in Omicron RBD-ACE2 structure was mutated to R493 to follow the recent sequence of Omicron. Therefore, two simulation systems, WT and Omicron were constructed. To keep the consistency among simulation systems, S19-D615 in ACE2 and T333-G526 in RBD were maintained in WT and the two Omicron systems.

FF19SB force field was applied to model the systems[6]. The initial structures were solvated in a truncated octahedral transferable intermolecular potential three point (termed as "TIP3P") water box with a buffer of 10 Å around it. Then, counterions Na+ or Cl- were added to the systems for neutralization and 0.15 mol·L^{-1} NaCl were added to solvents.

After construction, the systems were firstly minimized for 15,000 cycles with restraint of 500 kcal·mol^{-1}·Å^{-2} on the RBD and ACE2. Then, all atoms encountered 30,000 cycles of minimization. Next, the systems were heated from 0 to 300 K in 300 ps and equilibrated for 700 ps with 10 kcal·mol^{-1}·Å^{-2} positional restraint on non-solvent atoms. Finally, the WT, and Omicron simulation systems encountered 8 parallel rounds of 200 ns production MD simulations, respectively. The timestep during MD simulations is 2 fs. During simulations, the temperature (300 K) and pressure (1 atm) was controlled by Langevin thermostat and Berendsen barostat, respectively. Long-range electrostatic interactions were treated by Particle mesh Ewald algorithm with a grid size of 1 Å, and a cutoff of 10 Å was employed for short-range electrostatic and van der Waals interactions. The SHAKE algorithm was applied to restrain the bond with hydrogens. MD simulations were performed on Amber20, pmemd.cuda program.

Markov State Model (MSM)

Starting with the code base of the current stable version 3.8.0 of MSMBuilder[7], we developed a more robust algorithm to describe the transition process in Markov state model. Our algorithm modifies the fixed lag time into a random one by a kernel function, which is further used to count transition matrix and build MSM model. As a consequence, the MSM model based on our

algorithm exhibits a more robust and powerful representation ability for describing the protein conformational space. We will discuss this method in depth in our future publications.

From the trajectories of WT and Omicron system, the time-lagged independent component (tIC) analysis was firstly applied to decrease the dimension of the conformational space[8]. We selected the residue pairs, in which one residue was from RBD and the other was from ACE2. For each residue pair, we measured any pair of distances between the heavy atoms on RBD and those on ACE2 and kept the distances less than 5 Å as inputs for ContactFeaturizer. Then, the lag time of tIC analysis was set to 50 ns and 200 microstates were clustered by K-Centers algorithm. Multiple transition probability matrixes (TPMs) were further calculated according to the transitions among microstates. According to Eq. (1), the implied timescale test was performed to confirm the Markovian of microstates.

$$\tau_i = - \tau / \ln \lambda_i \quad (1)$$

where τ represents the lag time for the TPMs, λ_i is the i-th eigenvalue of the TPM and τ_i is the implied timescale for the i-th relaxation of the MSM. As a function of the lag time τ, τ_i (especially τ1 for the slowest transition) is a constant when the transition among microstates are Markovian[9,10]. From the Markovian microstates, the macrostates were then clustered via the PCCA+ algorithm. Using transition path theory (TPT), the properties for transition, such as transition time and direction, were calculated[11].

MM/GBSA calculation

MMPBSA.py plugin in AmberTools20 was applied to exploit Molecular Mechanics/Generalized Born Surface Area (MM/GBSA) in binding free energy calculations in balanced trajectories for WT, and Omicron simulation systems[12]. In total, the binding free energy (ΔG_{bind}) of RBD towards ACE2 is expressed as equation (2).

$$\Delta G_{bind} = G_{complex} - G_{ACE2} - G_{RBD} \quad (2)$$

Meanwhile, the second law of thermodynamics reflects that ΔG_{bind} equals to enthalpy changes (ΔH) minus the product of entropy changes and temperature (TΔS), as equation (3) expresses.

$$\Delta G_{bind} = \Delta H - T\Delta S \quad (3)$$

The system conformation entropy (termed as "−TΔS") is usually estimated by normal mode analysis with a quasi-harmonic model, however, accurate estimation of the conformation entropy for the protein-protein interactions remains challenging. Notably, the item could be omitted here considering that the differences of enthalpy (termed as "ΔH") are large enough and the similarity

of system conformation entropy among simulation systems[13]. Therefore, we omitted the calculation of the $-T\Delta S$ term and only concentrated on the relative ordering of the free energy changes.

In the simulation process, ΔH is transformed into the sum of the molecular mechanical energies (ΔE_{MM}) and the solvation free energy (ΔG_{solv}), according to equation (4).

$$\Delta H = \Delta E_{MM} + \Delta G_{solv} \quad (4)$$

In addition, ΔE_{MM} consists of the intramolecular energy (ΔE_{int} including bond, angle and dihedral energies), van der Waals energy (ΔE_{vdw}) and electrostatic energy (ΔE_{ele}), while ΔG_{solv} consists of the polar (ΔG_{P}) and the non-polar items (ΔG_{np}). Equations (5) and (6) represent them.

$$\Delta E_{MM} = \Delta E_{int} + \Delta E_{vdw} + \Delta E_{ele} \quad (5)$$

$$\Delta G_{solv} = \Delta G_{P} + \Delta G_{np} \quad (6)$$

The Generalized Born (GB) model was used to calculate ΔG_{P} with mbondi2 and GB^{OBC} model II. ΔG_{np} was calculated based on the solvent-accessible surface area (SASA) in equation (7). According to the GB model, SASA was calculated via LCPO and γ was 0.005.

$$\Delta G_{np} = \gamma SASA \quad (7)$$

The decomposition of the free energy into residues was subsequently carried out by the MMPBSA.py plugin[12]. During the decomposition process, 1-4 interactions were added to electric interactions and van der Walls interactions.

References

1 Minor, W., Cymborowski, M., Otwinowski, Z. & Chruszcz, M. *Acta crystallographica. Section D, Biological crystallography* **62**, 859-866, (2006).

2 McCoy, A. J. et al. *Journal of applied crystallography* **40**, 658-674, (2007).

3 Emsley, P. & Cowtan, K. *Acta crystallographica. Section D, Biological crystallography* **60**, 2126-2132, (2004).

4 Adams, P. D. et al. *Acta crystallographica. Section D, Biological crystallography* **58**, 1948-1954, (2002).

5 Janson, G., Zhang, C., Prado, M. G. & Paiardini, A. *Bioinformatics* **33**, 444-446, (2017).

6 Tian, C. et al. *Journal of chemical theory and computation* **16**, 528-552, (2020).

7 Harrigan, M. P. et al. *Biophysical journal* **112**, 10-15, (2017).

8 Naritomi, Y. & Fuchigami, S. *The Journal of chemical physics* **134**, 065101, (2011).

9 Swope, W. C. et al. *J Phys Chem B* **108**, 6582-6594, (2004).

10 Husic, B. E. & Pande, V. S. *J Am Chem Soc* **140**, 2386-2396, (2018).

11 Chodera, J. D., Singhal, N., Pande, V. S., Dill, K. A. & Swope, W. C. *Journal of Chemical Physics* **126**, (2007).

12 Miller, B. R. et al. *Journal of chemical theory and computation* **8**, 3314-3321, (2012).

13 Sun, H. Y. et al. *Phys Chem Chem Phys* **20**, 14450-14460, (2018).
